# High PIRCHE Scores May Allow Risk Stratification of Borderline Rejection in Kidney Transplant Recipients

**DOI:** 10.3389/fimmu.2022.788818

**Published:** 2022-02-18

**Authors:** Ekaterina Lezoeva, Jakob Nilsson, Rudolf Wüthrich, Thomas F. Mueller, Thomas Schachtner

**Affiliations:** ^1^ Department of Nephrology, University Hospital Zurich, Zurich, Switzerland; ^2^ Department of Immunology, University Hospital Zurich, Zurich, Switzerland

**Keywords:** borderline rejection, TCMR, ABMR, *de novo* DSA, epitope matching

## Abstract

**Background:**

The diagnosis of borderline rejection (BLR) ranges from mild inflammation to clinically significant TCMR and is associated with an increased risk of allograft dysfunction. Currently, there is no consensus regarding its treatment due in part to a lack of biomarkers to identify cases with increased risk for immune-mediated injury.

**Methods:**

We identified 60 of 924 kidney transplant recipients (KTRs) with isolated and untreated BLR. We analyzed the impact of predicted indirectly recognizable HLA epitopes (PIRCHE) score on future rejection, *de novo* DSA development, and recovery to baseline allograft function. Additionally, we compared the outcomes of different Banff rejection phenotypes.

**Results:**

Total PIRCHE scores were significantly higher in KTRs with BLR compared to the entire study population (p=0.016). Among KTRs with BLR total PIRCHE scores were significantly higher in KTRs who developed TCMR/ABMR in follow-up biopsies (p=0.029). Notably, the most significant difference was found in PIRCHE scores for the HLA-A locus (p=0.010). PIRCHE scores were not associated with the development of *de novo* DSA or recovery to baseline allograft function among KTRs with BLR (p>0.05). However, KTRs under cyclosporine-based immunosuppression were more likely to develop *de novo* DSA (p=0.033) than those with tacrolimus, whereas KTRs undergoing retransplantation were less likely to recover to baseline allograft function (p=0.003).

**Conclusions:**

High PIRCHE scores put KTRs with BLR at an increased risk for future TCMR/ABMR and contribute to improved immunological risk stratification. The benefit of anti-rejection treatment, however, needs to be evaluated in future studies.

## Introduction

The pathology diagnosis of borderline rejection (BLR) comprises various histologic lesions, ranging from mild inflammation to clinically significant T-cell mediated rejection (TCMR) (1). Adding further complexity, inflammation (i) and tubulitis (t) lesions can occur in the context of other graft injuries, such as ABMR, polyomavirus-nephropathy, and other infections, glomerulonephritis as well as acute kidney injury. The molecular mechanisms driving TCMR have been demonstrated to be responsible for developing the chronic histologic lesion of interstitial fibrosis/tubular atrophy (IF/TA) (2), which has been associated with graft failure (3, 4). Nankivell et al. showed that despite anti-rejection treatment, BLR was often followed by acute rejection episodes and increased *de novo* donor‐specific antibodies. At the same time, the rate of spontaneous resolution of inflammatory infiltrates without treatment was also high (5). However, even with the resolution of inflammatory changes in the subsequent biopsies, the BLR was associated with poorer allograft function and survival (5). Thus, the clinical significance of BLR is uncertain concerning the indication for therapy.

Only recently, the histopathologic criteria for the diagnosis of BLR have been investigated. Accordingly, the Banff classification was adjusted in 2019, the threshold for interstitial inflammation in BLR was changed to “interstitial inflammation involving 10%-25% of the non-sclerotic cortex (Banff i1) with at least mild tubulitis (Banff t>0)” (6). Contrary to the previous definition of BLR in the 2018 Banff Classification with the minimal lesion being “i0 t1”, the new definition of minimum lesion reads “i1 t1”.

In the past, there have been numerous efforts to develop new methods to diagnose potentially treatable graft injuries or predict future rejection risks. A comprehensive review of current research on immunologic and non-immunologic biomarkers was recently published by Swanson et al. (7) The innovative methods span across functional cell-based immune monitoring [IFN-γ-ELISPOT (8–10)], analysis of peripheral blood and urine for gene expression signatures by measuring mRNA (11–13), microRNA (14, 15), and dd-cfDNA (16, 17), as well as molecular phenotyping of graft biopsies [molecular microscope (MMDx)] (18), which is emerging as a promising new tool in biopsy interpretation.

However, predictive biomarkers of BLR have not been adequately studied, and there are no established histologic or immunologic criteria to stratify the risk in diagnosis and treatment of BLR. Furthermore, there are no consensus clinical guidelines for BLR, and the long-term consequences of detecting and treating subclinical inflammation remain controversial (19–21). Thus, the clinical community is in dire need of a marker to identify those cases with BLR that result in immune-mediated injury, potentially leading to adverse immunological, function, and histological events. Recently, measurement of dd-cfDNA has been proposed as a complement marker for risk stratification in BLR (22) and is under investigation in an ongoing trial in combination with MMDx (Halloran PF, INTERCOMEX DD-cfDNA-HLA-MMDx Study: Comparing the DD-cfDNA Test to MMDx Microarray Test, Central HLA Antibody Test, and Histology. 2021. In Proceedings from https://clinicaltrials.gov/ct2/show/NCT04239703).

The risk of rejection is linked to HLA matching. The impact of individual HLA mismatches between donor and recipient on alloreactivity is strongly influenced by the amount of immunogenic allo epitopes. Based on the mismatched donor HLA type, the Predicted Indirectly Recognizable HLA Epitopes (PIRCHE) score estimates the number of indirectly recognizable, donor HLA derived T cell epitopes and predicts T cell-related immune responses against the donor HLA-derived peptides. The PIRCHE score - as a marker for the allo-immunogenicity of donor-recipient HLA mismatch – was shown to be associated with the risk for *de novo* (dnDSA) occurrence and long-term kidney allograft survival in two large kidney transplant cohorts (23, 24). Very recently, our own data showed, that high PIRCHE scores were associated with an increased risk of rejection among KTRs with low-level BKV-DNAemia (25). Being a novel method to better assess the risk for cellular rejection, the PIRCHE score has not been investigated so far as a biomarker in BLR. In the present study, we attempted to address the following questions: (1) Do HLA T-cell epitope mismatches predict acute rejection (TCMR and ABMR) in subsequent biopsies in KTRs with BLR? (2) Do HLA T-cell epitope mismatches predict recovery of allograft function in KTRs with BLR? (3) Do HLA T-cell epitope mismatches predict the development of dnDSA in KTRs with BLR? (4) Is there a difference regarding the clinical outcome in BLR depending on the applied histologic diagnostic criteria of threshold lesion “i0 t1” vs. “i1 t1” (Banff classification 2018 vs. Banff classification Update 2019)?

## Materials and Methods

### Patients

Our study was approved by the cantonal ethic commission review board of Zurich, Switzerland (KEK-ZH-Number 2020-02817) and has been conducted in compliance with the declaration of Helsinki.

**Figure 1:**
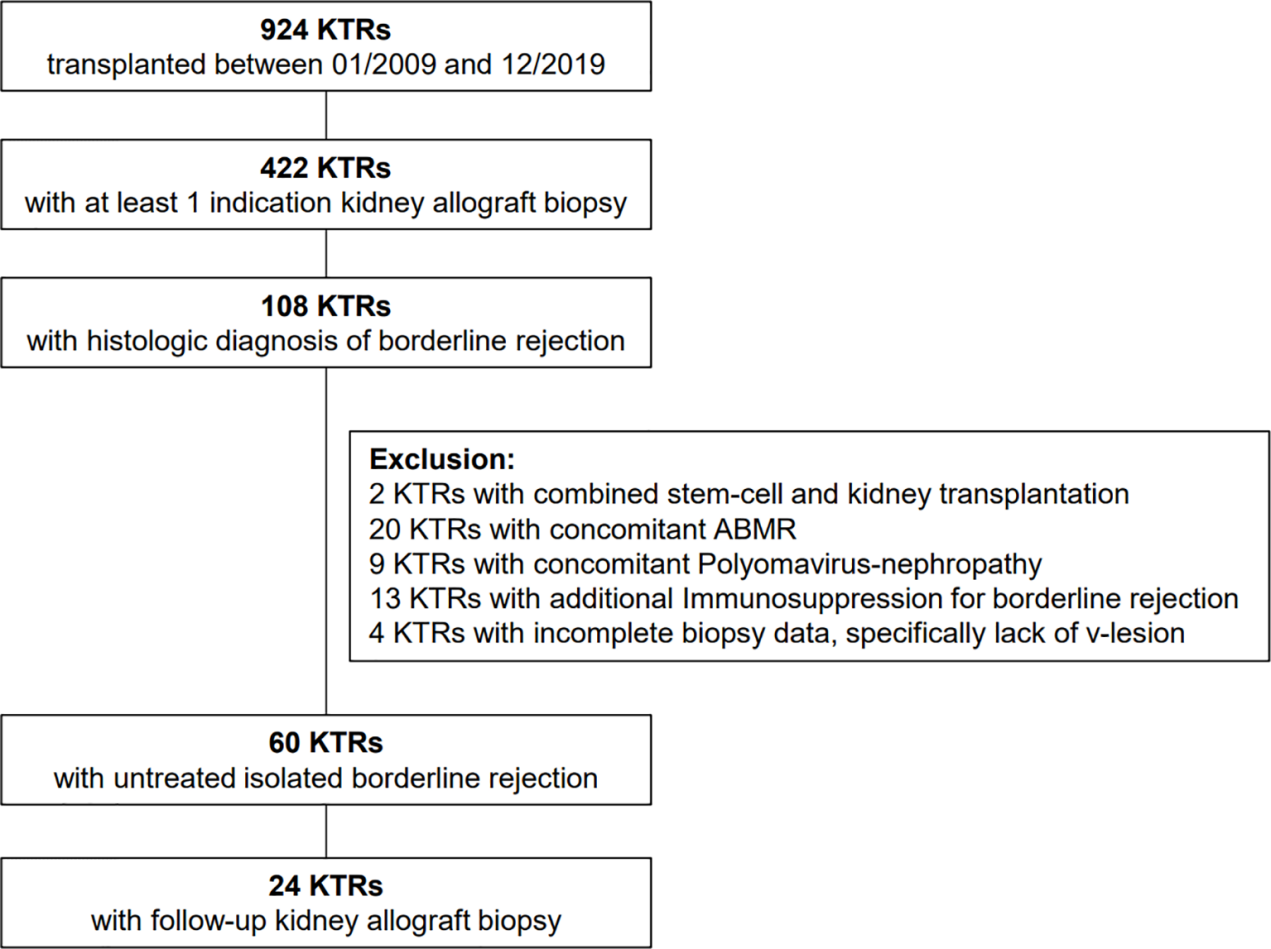
Patient inclusion and exclusion algorithm.

We performed a retrospective study of all 924 KTRs who underwent kidney transplantation at the University Hospital of Zurich between January 1, 2009, and December 30, 2019. During the follow-up period of at least one year, 422 KTRs received at least one kidney allograft biopsy. No biopsies during delayed graft function (DGF) were included in this analysis. From this cohort, we selected 108 KTRs with the diagnosis of BLR according to the Banff classification of 2018. Only KTRs who were not treated for BLR were included in the analysis. In total, 48 KTRs were excluded from the analysis: 2 KTRs with combined stem cell transplantation, 20 KTRs with concomitant ABMR, 13 KTRs treated with specific immunosuppressive therapy for TCMR or any other reason (relapse of FSGS, relapse of MPGN, suspected rejection prior to biopsy), 9 KTRs with polyomavirus nephropathy, and 4 KTRs with insufficient biopsy quality, specifically lack v-lesion score (**Figure 1**). All KTRs had a minimum follow-up period of one year. Further progression was evaluated in terms of (1) occurrence of clinically relevant rejection (TCMR ≥1A or/and ABMR) within 12 months after the first biopsy, (2) significant deterioration of allograft function (defined as an increase of baseline creatinine >20% during the 1^st^ year after diagnosis of BLR), and (3) development of dnDSA.

Post-transplant care was carried out according to a standardized scheme with appointments in the outpatient clinic twice a week at week 2 and 3, at week 4, 5, 6, 8, 10, 12, at month 4, 5, 6, 8, 10 and 12, with at least 16 visits within the first year after transplantation. Subsequently, quarterly check-ups were performed in cooperation with the nephrologists close to the patient’s home, with at least annual follow-up visits in our outpatient clinic. Screening for CMV-DNAemia was performed according to the CMV risk status of the KTR. Screening for BKV-DNAemia was conducted monthly in the first six months, at months 8, 10, 12, and 18, and any unclear deterioration of kidney allograft function. The anti-HLA antibody testing was performed with use of a Luminex based assay (One Lambda, Canoga Park, CA, USA), on the day of transplantation, at months 3, 6, 12, and annually after that, and at any other time in case of unexplained deterioration of allograft function.

### Induction and Maintenance Immunosuppression

The choice of induction therapy was based on immunological risk. KTRs with a low-immunologic risk (no anti-HLA antibodies with MFI >1000 at any time prior to transplantation) received IL2-receptor blockade with basiliximab, and KTRs with a high-immunologic risk (anti-HLA antibodies with MFI >1000 at any time prior to transplantation or any DSA with MFI <1000) received lymphocyte-depleting induction with thymoglobulin. No transplantation with DSA and MFI >1000 are performed.

ABO desensitization included a single dose of rituximab before transplantation and blood group-specific immunoadsorption. The primary immunosuppression consisted of a triple-drug combination of a calcineurin inhibitor (CNI), tacrolimus or cyclosporine, antimetabolite (mycophenolate mofetil (MMF) or mycophenolic acid (MPA) or azathioprine), and steroids. The initial dose of tacrolimus was 0.2 mg/kg body weight/day, and trough levels were maintained at 10-15µg/l until week 6, at 8-12 µg/l until week 12, at 7-10 µg/l until month 12, at 6-8µg/l until month 24, and at 4-6 µg/l after that. The initial dose of cyclosporine was 8mg/kg body weight, and target trough levels were at 200-250 µg/l until week 6, at 180-220 µg/l until week 12, at 150-200 µg/l until month 12, at 80-120 µg/l until month 24, and at 60-100 µg/l after that. The dosage of MMF was 2000 mg/day, and the dosage of MPA was 1440 mg/day. Steroid tapering was performed over 12 weeks to a dose of 5 mg prednisone/day. According to immunologic risk, steroid withdrawal was implemented.

### Assessment of Kidney Allograft Function and Kidney Allograft Biopsies

To evaluate the kidney allograft function, we compared the serum-creatinine baseline before the biopsy with the finding of BLR, with the increase in serum-creatinine that led to the biopsy and the creatinine baseline one year after the biopsy. The creatinine baseline was calculated as the median of 3 lowest creatinine values for both periods, respectively.

In total, 60 indication biopsies and 24 follow-up indication biopsies were included in the analysis. The biopsies were evaluated by an experienced renal pathologist, not blinded to clinical information. The rejection was classified according to the Banff 2018 reference guide (6). The scores for tubulitis (t) were defined by the maximum number of mononuclear cells per tubular cross-section or ten tubular epithelial cells as t0 for 0, t1 for 1–4, t2 for 5–10, and t3 for>10, in not severely atrophic tubules. The scores for inflammation in non-scarred cortex (i) were defined by a degree of inflammation as i0 for<10%, i1 for 10%–25%, i2 for 26%–50%, and i3 for>50%. A diagnosis of BLR required at least t1 and i0.

### Calculation of Predicted Indirectly ReCognizable HLA Epitopes (PIRCHE)

The HLA-derived mismatched peptide epitopes presented by KTR’s HLA-molecules were calculated using the PIRCHE algorithm. Presentation of both HLA class I (HLA-A, B, C) and HLA class II derived peptides (HLA-DR, DQ) were calculated for each HLA locus, and designated PIRCHE-A, B, C, DR, and DQ. HLA typing of KTRs was achieved by serological and DNA-based techniques. The PIRCHE algorithm is available online (https://www.pirche.org).

### Statistical Methods

Statistical analysis was performed using IBM SPSS Version 25 (SPSS, Chicago, IL, USA). For comparisons of study groups, Mann–Whitney U-Test was used for nonparametric independent samples. For comparisons between paired samples, a two-sided Wilcoxon signed-rank test for nonparametric dependent samples was used. Multiple linear regression models were used to investigate independent risk factors. -Clinical characteristics were compared across groups using Fisher’s exact test for categorical variables. Boxplots show median, interquartile range (IQR), and 95^th^ percentile.

## Results

### Overall Patient Characteristics

**Figure 2:**
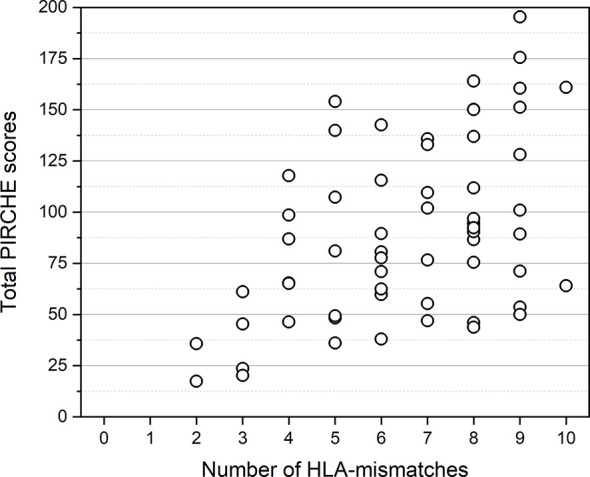
Distribution of total PIRCHE scores compared to total HLA-mismatches. PIRCHE scores and the number of HLA-mismatches were calculated from HLA class I (HLA-A, B, C) and HLA class II (HLA-DR, DQ) mismatches. Median PIRCHE scores for 4, 5, 6, 7, 8, 9, and 10 HLA-mismatches were 76.15, 81.07, 77.63, 101.97, 94.66, 114.56, and 112.45, respectively.

From the cohort of 924 KTRs transplanted between January 2009 and December 2019, we identified 60 KTRs with BLR in a first indication biopsy, who fulfilled the inclusion criteria (**Figure 1**). Clinical characteristics and biopsy characteristics are shown in **Tables 1A**, **B**. The median total PIRCHE score of 60 KTRs with BLR was 86.78 (range: 17.37-195.43) with PIRCHE-A of 14.36 (0-69.37), PIRCHE-B of 15.12 (0-44.63), PIRCHE-C of 13.64 (0-37.09), PIRCHE-DR of 14.77 (0-56.13), and PIRCHE-DQ of 23.00 (0-60.13; **Figure 2**). The median total PIRCHE score of the remaining study population of 864 KTRs was 71.99 (range: 0-286.78) with PIRCHE-A of 14.53 (0-69.37), PIRCHE-B of 14.28 (0-72.72), PIRCHE-C of 12.93 (0-75.06), PIRCHE-DR of 12.47 (0-74.78), and PIRCHE-DQ of 19.00 (0-82.55). The 60 KTRs with BLR showed higher total PIRCHE scores compared to the remaining study population of 864 KTRs (p=0.016).

**Table 1A:** Clinical characteristics of KTRs with/without acute rejection in follow-up biopsies.

	Total (n = 60)	No Rejection (n = 52)	Rejection (n = 8)	*P value*
**Recipient Characteristics**				
Recipient age, years*	56.5 (19-74)	55.5 (19-74)	62 (53-72)	*0.157*
Renal disease, n (%)				
Diabetic	7 (11.7%)	7 (13.5%)	0	*0.424*
Hypertensive	4 (6.6%)	3 (5.8%)	1 (12.5%)	
PKD	7 (11.7%)	7 (13.5%)	0	
Glomerular disease	24 (40%)	21 (40.4%)	3 (37.5%)	
Others/unknown	18 (30%)	14 (26.9%)	4 (50%)	
Recipient, male sex, n (%)	35 (58.3%)	31 (59.6%)	4 (50%)	*0.708*
Deceased donation, n (%)	46 (76.7%)	38 (73.1%)	8 (100%)	*0.180*
Living donation, n (%)	14 (23.3%)	14 (26.9%)	0	*0.180*
AB0 incompatible, n (%)	2 (3.3%)	2 (3.8%)	0	*1*
Retransplantation, n (%)	14 (23.3%)	13 (25%)	1 (12.5%)	*0.666*
Cold ischemia time, minutes*	565 (312-1197)	565 (316-1197)	561 (312-1195)	*0.954*
**Immunosuppression**				
Induction IS, n (%)				*1*
Lymphocyte depletion	23 (38.3%)	20 (38.5%)	3 (37.5%)	
IL-2 receptor blockade	34 (56.7%)	29 (55.8%)	5 (62.5%)	
AB0 desensitization	2 (3.3%)	2 (3.8%)	0	
Other	1 (1.7%)	1 (1.9%)	0	
Maintenance IS, n (%)				
Tacrolimus	49 (81.7%)	44 (84.6%)	5 (62.5%)	*0.154*
Ciclosporine	11 (18.3%)	8 (15.4%)	3 (37.5%)	*0.154*
MMF/MPA	59 (98.3%)	51 (98.1%)	8 (100%)	*1*
Azathioprin	1 (1.7%)	1 (1.9%)	0	*1*
Steroid free at 1 year, n (%)	28 (46.7%)	23 (44.2%)	5 (62.5%)	*0.454*
**Donor Characteristics**				
Donor age, years*	56 (0-78)	54.5 (0-75)	65 (45-78)	*0.054*
Donor male sex n (%)	29 (48.3%)	24 (46.15%)	5 (62.5%)	*0.465*
**Immunocompatibility**	7 (2-10)			
Total HLA Mismatches*	5.5 (1-10)	7 (2-10)	8 (5-9)	*0.367*
Total PIRCHE-Score*	86.78 (17.37-195.43)	79.10 (17.37-195.43)	124.40 (62.52-175.62)	0.029*
PIRCHE-A*	14.36 (0-69.37)	12.98 (0-69.37)	31.15 (11.27-51.71)	*0.0099*
PIRCHE-B*	15.12 (0-44.63)	13.61 (0-44.63)	21.33 (8.85-40.80)	*0.177*
PIRCHE-C*	13.64 (0-37.09)	13.64 (0-37.09)	16.24 (5.44-31.93)	*0.564*
PIRCHE-DR*	14.77 (0-56.13)	13.81 (0-56.13)	17.84 (6.47-34.0)	*0.447*
PIRCHE-DQ*	23.00 (0-60.13)	22.84 (0-55.72)	33 (15.11-60.13)	*0.097*
Preformed DSA, n (%)^§^	18 (30%)	16 (30.8%)	2 (25%)	*1*

*median (range).

^§^Preformed DSA: DSA against the current renal graft with MFI > 500 at any time before the transplantation, but latest MFI <1000 prior to transplantation.

**Table 1B:** Outcomes of KTRs of KTRs with/without acute rejection in follow-up biopsies.

	Total (n = 60)	No Rejection (n = 52)	Rejection (n = 8)	*P value*
DGF, n (%)	12 (20%)	10 (19.2%)	2 (25%)	*0.655*
**First allograft biopsy**				
Time after transplantation, months*	4.5 (0-74)	4.5 (0-74)	4 (0-58)	*1*
< 6 months, n (%)	34 (56.7%)	29 (55.8%)	5 (62.5%)	*0.183*
6-12 months, n (%)	11 (18.3%)	11 (21.2%)	0	
13 - 60 months, n (%)	13 (21.7%)	10 (19.2%)	3 (37.5%)	
> 60 months, n (%)	2 (3.3%)	2 (3.8%)	0	
Indication for biopsy				
Protocol, n (%)	3 (5%)	3 (5.8%)	0	*0.480*
eGFR/Proteinuria, n (%)	52 (86.7%)	45 (86.5%)	7 (87.5%)	
BKV replication, n (%)	2 (3.3%)	2 (3.8%)	0	
DSA, n (%)	3 (5%)	2 (3.8%)	1 (12.5%)	
**Biopsy Findings**				
Borderline only, n (%)	16 (26.7%)	14 (26.9%)	2 (25%)	*0.975*
Borderline and ATN, n (%)	15 (25%)	13 (25%)	2 (25%)	
Borderline and arteriolar hyalinosis, n (%)	5 (8.3%)	4 (7.7%)	1 (12.5%)	
Borderline and other^†^, n (%)	24 (40%)	21 (40.4%)	3 (37.5%)	
i0 t≥1, n (%)	51 (85%)	44 (84.6%)	7 (87.5%)	*1*
i0 t1, n (%)	43 (71.7%)	36 (69.2%)	7 (87.5%)	*0.272*
i0 t2, n (%)	5 (8.3%)	5 (9.6%)	0	
i0 t3, n (%)	3 (5%)	3 (5.8%)	0	
i≥1 t≥1, n (%)	9 (15%)	8 (13.5%)	1 (12.5%)	*1*
i1 t1, n (%)	4 (6.7%)	4 (7.7%)	0	*0.405*
i1 t2, n (%)	3 (5%)	2 (3.8%)	1 (12.5%)	
i1 t3, n (%)	1 (1.7%)	1 (1.92%)	0	
i2 t1, n (%)	0	0	0	
i3 t1, n (%)	1 (1.7%)	1 (1.92%)	0	
***De novo* DSA, n (%)^#^**	12 (20%)*	9 (17.3%)	3 (37.5%)	*0.191*
Time after first biopsy, months*	22 (3-48)	24 (8-48)	20 (3-35)	*0.255*
IgG Class I, n (%)	4 (6.7%)	3 (5.8%)	1 (12.5%)	*-*
IgG Class II, n (%)	9 (15%)	6 (11.53%)	3 (37.5%)	*-*
**Viral infections**				
BKV replication at any time, n (%)	16 (26.7%)	14 (26.9%)	2 (25%)	*1*
BKV replication at time of biopsy, n (%)	12 (20%)	11 (21.2%)	1 (12.5%)	*1*
CMV replication at any time, n (%)	34 (56.7%)	27 (51.9%)	7 (87.5%)	*0.122*
CMV replication at time of biopsy, n (%)	11 (18.3%)	9 (17.3%)	2 (25%)	*0.631*
**Follow-up biopsy**				
Time after first biopsy, months*	4 (0-12)	5 (0-12)	3 (1-10)	*0.265*
Rejection in follow-up biopsy, n (%)	8 (13.3%)	–	8 (100%)	*-*
TCMR 1A and 1B, n (%)	1 (1.7%)	–	1 (12.5%)	*-*
TCMR 2A and 2B, n (%)	6 (10%)	–	6 (75%)	*-*
ABMR, n (%)	1 (1.7%)	–	1 (12.5%)	*-*

*median (range).

^†^Other: recurrence of renal disease (1 KTR with FSGS, 3 KTRs with IgAN), 3 KTRs with CNI-associated toxicity, 1 KTR with interstitial fibrosis, 2 KTRs with TMA, 2 KTRs with necrotic lesions, 2 KTRs with obstruction, 3 KTRs with pyelonephritis, 1 KRT with adenovirus-associated acute tubulointerstitial nephritis, 1 KTR with oxalate crystals, 2 KTRs with IgA-positivity with unclear clinical significance, 1 KTR with presumable Foscarnet-associated changes.

^#^1 KTR has developed de novo DSA for both class I and II.

Among the 60 KTRs, BLR was the only pathologic finding in 16 KTRs (26.7%). BLR was accompanied by other histologic changes, namely acute tubular necrosis (ATN) in 15 KTRs, arteriolar hyalinosis in 5 KTRs, recurrence of renal disease in 4 KTRs, CNI-associated toxicity in 3 KTRs, and pyelonephritis in 3 KTRs. In 2 KTRs the additional findings were TMA and IgA-positivity of unclear clinical significance.

### Impact of PIRCHE Scores on Acute Rejection in Follow-Up Biopsies

Within 12 months after the diagnosis of BLR, allograft rejection occurred in the follow-up biopsies of 8 KTRs. The majority of cases (n=7) were classified as TCMR, while ABMR was reported in one KTR. Clinical and biopsy characteristics are shown in **Tables 1A**, **B**.

**Figure 3:**
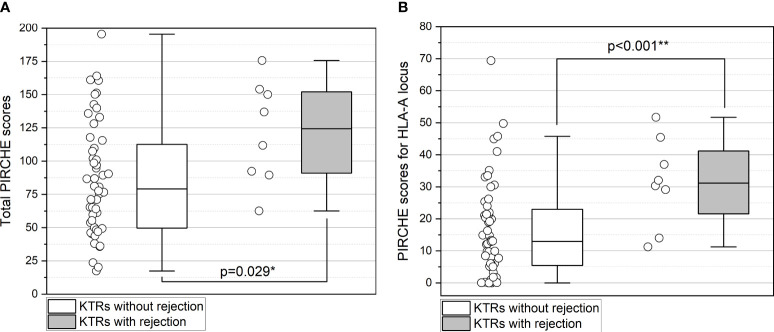
**(A, B)** Higher total PIRCHE scores **(A)** and PIRCHE scores for HLA-A locus mismatches **(B)** among KTRs with BLR who develop future TCMR/ABMR. Boxplots show median, interquartile range (IQR), and 95^th^ percentile. ** significance level p<0.01.

The median total PIRCHE score was significantly higher in KTRs with subsequent rejection with a median of 124.40 (range 62.52-175.62; p=0.029; **Figure 3A**). Notably, among the individual PIRCHE scores, the most significant difference was found in the median PIRCHE-A with 31.15 (range 11.27-51.71) in KTRs with rejection compared to 12.98 (range 0-69.37) without rejection (p=0.001; **Figure 3B**) and less pronounced in the median PIRCHE-DQ with 33.00 (15.11-60.13) compared to 22.84 (0-55.72; p=0.097). Upon multivariate analysis, PIRCHE-A remained the only independent risk factor for the development of acute rejection on follow-up biopsy (p=0.010). The PIRCHE values and distribution range are illustrated in **Figure 3**. KTRs with rejection showed a higher incidence of dnDSA at 37.5% compared to 17.3% in KTRs without rejection. Concerning allograft function one year after the first biopsy, no significant differences were observed between the two groups (p=0.136).

### Impact of PIRCHE Scores on Recovery of Allograft Function

Of the 60 KTRs with BLR, 23 KTRs (38.3%) did not recover to baseline creatinine within one year after the indication biopsy. Instead, they showed serum-creatinine values >20% compared to baseline before the biopsy indicating BLR. Clinical and biopsy characteristics are shown in **Supplement Tables 1A, B**. The median total PIRCHE score and individual scores for PIRCHE-A, B, C, DQ, and DR did not differ between the two groups (p>0.05; **Supplement Tables 1A, B**). At the same time, we observed that KTRs who did not recover to baseline creatinine more likely underwent re-transplantation (p=0.005) and more often had preformed DSA (p=0.023). Upon multivariate analysis, retransplantation remained the only independent risk factor not to recover to baseline creatinine (p=0.003). In addition, in 23 KTRs who did not recover to baseline allograft function, the time to biopsy was longer (median 14 months vs. two months), and arteriolar hyalinosis was a more frequently described incidental finding (17.5% vs. 2.7%).

### Impact of PIRCHE Score on the Development of dnDSA

Among the 60 KTRs, dnDSA was detected in 12 KTRs (20%). Clinical and biopsy characteristics are shown in **Supplement Tables 2A, B**. The median total PIRCHE score and individual scores for PIRCHE-A, B, C, DQ, and DR did not differ between the two groups (p>0.05; **Supplement Table 2A**). Notably, a more significant proportion of KTRs with dnDSA received cyclosporine-based immunosuppression (41.2%) compared to those who did not develop dnDSA (12.5%; p=0.033).

### Impact of the Banff Phenotype “i0 t1” *vs*. “i1 t1” as the Threshold Lesion

In our cohort, the initial 60 and 24 follow-up biopsies were assessed according to the current Banff classification. The Banff Update of 2019, which proposed the reclassification of the threshold lesion for BLR as “i1 t1,” was published in May 2020. With the last biopsy performed on 27.02.2020, the previous Banff classification used was that of 2018. Among the 60 KTRs, only 9 KTRs (15%) had an “i”-lesion ≥1 and fulfilled the new definition of BLR. Clinical and biopsy characteristics are shown in **Supplement Tables 3A, B**.

**Figure 4:**
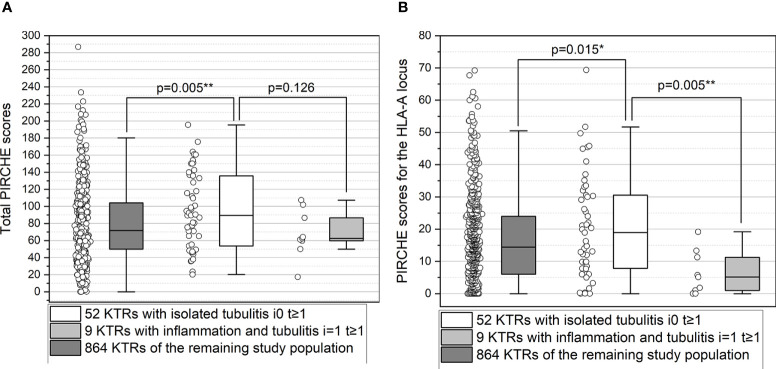
**(A, B)** Higher total PIRCHE scores **(A)** and PIRCHE scores for HLA-A locus mismatches **(B)** among KTRs with isolated tubulitis compared to KTRs with inflammation and tubulitis. Boxplots show median, interquartile range (IQR), and 95^th^ percentile. * significance level p<0.05; ** significance level p<0.01.

The median total PIRCHE score did not differ between the two groups (p>0.05; **Supplement Tables 3A, B**). Interestingly, KTRs with isolated tubulitis (i0 t≥1) showed higher PIRCHE scores for the HLA-A locus (p=0.005; **Figure 4**), higher PIRCHE scores for the HLA-B locus (p=0.014), and higher donor age (p=0.010) compared to KTRs with inflammation and tubulitis (“i≥1 t≥1”). Upon multivariate analysis, PIRCHE-A (p=0.027) and donor age (p=0.031) remained the only independent factors associated with “i0 t≥1” vs. “i≥1 t≥1”. KTRs with i0 t≥1 showed higher total PIRCHE scores (p=0.0054) and higher PIRCHE scores for the HLA-A locus (p=0.015) compared to the remaining study population of 864 KTRs. No differences were observed between KTRs with i≥1 t≥1 compared with the remaining study population of 864 KTRs (p>0.05; **Figures 4A, B**).

## Discussion

The diagnosis of BLR involves a heterogeneous group of histologic changes in the allograft and is associated with an adverse functional outcome and an increased risk of rejection (26). The diagnostic criteria for BLR have been changed in the last Banff update (2019) to improve risk stratification (6). However, the response to therapy is sometimes inconsistent even in clinically significant TCMR (27), and consensus guidelines regarding treatment for BLR are lacking. The results of commonly accepted treatment strategies have been shown in the recent comprehensive UNOS survey from kidney transplant programs in the USA (28). The challenge to balance adequate therapy to prevent allosensitization while avoiding over-immunosuppression often places clinicians at difficult management decisions. Recent research has been striving to find possible methods for risk stratification.

In the last years, dd-cfDNA has emerged as a novel, promising biomarker to assess the probability of acute rejection. Plasma levels of dd-cfDNA were repeatedly shown to correlate with allograft rejection (29). In a recent multicenter analysis, Stites et al. found that in KTRs with BLR and TCMR 1A, elevated levels of dd-cfDNA >0.5% were predictive of a more significant decline in GFR and a higher incidence of dnDSA, as well as further episodes of rejection (22). According to previous studies, however, dd-cfDNA levels were more reliable to discriminate ABMR and had a limited performance in detecting TCMR (30). However, dd-cfDNA is also released in non-alloimmune processes associated with allograft injury or systemic conditions. As such, increased levels of dd-cfDNA are not specific for any form of rejection, including TCMR.

HLA epitope matching algorithms presumably offer a more precise assessment of HLA compatibility and have lately enhanced the conventional molecular donor/recipient HLA mismatch calculation. In addition, PIRCHE is supposed to estimate the immunological potential for indirect CD4+ T-cell alloreactivity – a mechanism, which is thought to be crucial in the progression of alloreactivity and rejection in organ transplantation. Finally, the cohort analysis of more than 65,000 KTRs from the Collaborative Transplant Study suggested that PIRCHE scores might be a strong predictor of 5-year death-censored graft loss (31).

Inherent to the molecular principle of HLA-epitope recognition in PIRCHE, it is highly likely that high PIRCHE scores are associated with the development of circulating donor-specific T cells and subsequent TCMR (32, 33). Taking these observations into account, our study has sought to evaluate the correlation between PIRCHE scores and the prognosis of BLR. To our knowledge, this is the first study to investigate the impact of PIRCHE scores as a biomarker for the risk stratification in BLR.

Firstly, we observed the highest PIRCHE scores in KTRs with BLR who experienced clinically relevant rejection, primarily acute TCMR in subsequent biopsies. This finding suggests that PIRCHE scores could have possible utility as a prognostic biomarker for risk assessment in KTRs with BLR. Since in the early phase after transplantation the diagnosis of BLR may be further complicated by other histopathological changes such as ATN, the PIRCHE score appears to be of even greater importance as a biomarker for risk stratification of T-cell alloreactivity. In our study, the incidence of rejection was associated with disproportionately higher PIRCHE scores for HLA class I. These findings support observations that early acute rejection result from CD4 T-cell dependent cytotoxic CD8 T cell responses directed against HLA-class I allo-antigens generated by indirect pathway presentation (34). This finding contrasts with that by Wiebe et al., who showed a strong correlation between HLA‐DR/DQ molecular mismatch category and TCMR, including BLR (35). However, Wiebe et al. used HLA Matchmaker for the analysis. The two methods of HLA epitope matching follow different principles, making direct comparison difficult. Tomosugi et al. showed only a moderately positive correlation between the PIRCHE and the HLA Matchmaker scores in individual recipient and donor pairs (36). More importantly, the HLA Matchmaker algorithm predicts epitopes (eplets) involved in the humoral immune response, and only epitopes that are accessible to HLA antibodies are considered eplets. In contrast, the PIRCHE algorithm estimates the number of indirectly recognizable T cell epitopes and predicts T cell-related immune responses. Therefore, the PIRCHE algorithm appears more reasonable to stratify the risk of T-cell mediated injury as in BLR and TCMR (37).

Secondly, in our study of KTRs with BLR, PIRCHE scores were not associated with the development of dnDSA. This finding is inconsistent with current research that the number of PIRCHE is linked to an increased risk for the development of dnDSA (24). However, PIRCHE scores have not been evaluated in a cohort of KTRs with clearly defined BLR so far. In addition, follow-up time may have been too short for adequately investigating the development of dnDSA. Notably, a significantly greater proportion of KTRs with dnDSA received cyclosporine as maintenance immunosuppression, which may have contributed to this finding. This is in line with the current body of experience since the pivotal Symphony trial, which demonstrated superior outcomes in terms of both acute rejection rates and GFR for KTRs with tacrolimus-based regimen compared with cyclosporine- or sirolimus-based maintenance immunosuppression (38). Thus, although some experts recommend increasing maintenance immunosuppression in KTRs with BLR, a switch to a tacrolimus-based regime appears reasonable concerning our findings.

Thirdly, we observed that KTRs who did not recover to baseline allograft function were more likely to have preformed DSA. These findings at least suggest an ongoing smoldering immune-related injury that may benefit from anti-rejection treatment. Finally, KTRs with preformed allosensitization have been shown to have alloreactive T-cells in addition to alloreactive antibodies (10, 32). These preformed alloreactive T-cells may be responsible for the observed decline in kidney allograft function.

Fourthly, KTRs with isolated tubulitis (“i0,t≥1”) showed higher total PIRCHE scores and higher PIRCHE scores for the HLA-A locus compared to the entire study population and compared to KTRs with inflammation and tubulitis (“i≥1 t≥1”). This finding is highly interesting. Although the recent Banff 2019 update suggests that isolated tubulitis is no longer included in the category of BLR (6), our findings strongly show, that isolated tubulitis in KTRs who underwent an indication biopsy, mostly for deterioration of kidney allograft function, is associated with a biomarker for T-cell alloreactivity. These findings suggest that isolated tubulitis may not be unspecific, at least in a subgroup of KTRs, but may represent an immune-related injury pathophysiologically based on the high number of HLA-epitope mismatches. Our findings show that the subgroup with the highest number of HLA-epitope mismatches is at the greatest risk for progression to TCMR/ABMR. Although, Wiebe et al. very recently reported no difference in dnDSA free survival or allograft survival in KTRs with isolated Banff i0 t1 phenotype versus KTRs without tubulitis, our study suggests PIRCHE as a new biomarker to identify those KTRs with isolated tubulitis at increased risk for immune-related injury (35).

Our finding that KTRs with isolated tubulitis but not inflammation and tubulitis are identified by higher PIRCHE scores suggests, that the immune-related injury in these KTRs (“i≥1 t≥1”) may be based on other risk factors than high numbers of HLA-epitope mismatches. These risk factors may particularly involve preformed donor-reactive T-cells that have been detected in almost one third of all KTRs (39) or insufficient immunosuppression.

Our study has a few limitations, the main being the lack of protocol biopsies in most KTRs. Future studies should investigate the influence of the PIRCHE score especially in KTRs with subclinical BLR also in the context of protocol biopsies, as the data situation and recommendations regarding therapy are even more unclear in these cases. Secondly, the cohort size was comparatively small with limited number of events, and the hypothesis needs to be validated in a much larger cohort. Thirdly, the results might have been limited by retrospective design and single‐center approach.

Our study has several strengths. We included excellently characterized KTRs over ten years. A standardized immunosuppressive protocol and close functional/clinical monitoring post-transplant enabled us to obtain a very high data density. The kidney biopsies were read by the same renal pathologist, which minimized the interpersonal variability of histopathologic evaluation and uniformly scored by the Banff 2018 classification.

In summary, it is imperative to identify those KTRs with BLR who are at increased risk for future immunologic events and who would potentially benefit from additional immunosuppressive therapy. The principal findings of our study are that KTRs with BLR show higher PIRCHE scores compared to the entire study population and that among KTRs with BLR a higher PIRCHE score is associated with rejection in follow-up biopsies. Thus, total PIRCHE scores may contribute to improved immunological risk stratification and help to personalize management in KTRs with BLR.

## Data Availability Statement

The raw data supporting the conclusions of this article will be made available by the authors, without undue reservation.

## Ethics Statement

The studies involving human participants were reviewed and approved by Cantonal ethic commission review board of Zurich, Switzerland (KEK-ZH-Number 2020-02817). Written informed consent for participation was not required for this study in accordance with the national legislation and the institutional requirements.

## Author Contributions

EL participated in data collection, data analysis, and writing of the paper. JN participated in data collection and writing of the paper. RW participated in writing of the paper. TM participated in writing of the paper. TS participated in research design, data collection, data analysis, writing of the paper. All authors contributed to the article and approved the submitted version.

## Conflict of Interest

The authors declare that the research was conducted in the absence of any commercial or financial relationships that could be construed as a potential conflict of interest.

## Publisher’s Note

All claims expressed in this article are solely those of the authors and do not necessarily represent those of their affiliated organizations, or those of the publisher, the editors and the reviewers. Any product that may be evaluated in this article, or claim that may be made by its manufacturer, is not guaranteed or endorsed by the publisher.

## Abbreviations

ABMR, antibody-mediated rejection; BKV, polyomavirus BK; BLR, borderline rejection; CMV, cytomegalovirus; CNI, calcineurin inhibitor; dd-cfDNA, donor-derived cell-free DNA; DGF, delayed graft function; dnDSA, *de novo* donor-specific anti HLA-antibodies; GFR, glomerular filtration rate; HLA, human leukocyte antigen; KTRs, kidney transplant recipients; MMDx, molecular microscope diagnostic system; MMF, mycophenolate mofetil; MPA, mycophenolic acid; PIRCHE, Predicted Indirectly ReCognizable HLA Epitopes; TCMR, T-cell mediated rejection; TMA, thrombotic microangiopathy.

## References

[1] Roufosse C Simmonds N Clahsen-van Groningen M Haas M Henriksen KJ Horsfield C. A 2018 Reference Guide to the Banff Classification of Renal Allograft Pathology. Transplantation (2018) 102(11):1795–814. doi: 10.1097/TP.0000000000002366 PMC7597974 30028786

[2] Lefaucheur C Gosset C Rabant M Viglietti D Verine J Aubert O. T Cell-Mediated Rejection is a Major Determinant of Inflammation in Scarred Areas in Kidney Allografts. Am J Transplant (2018) 18(2):377–90. doi: 10.1111/ajt.14565 29086461

[3] Nankivell BJ Shingde M Keung KL Fung CL Borrows RJ O'Connell PJ. The Causes, Significance and Consequences of Inflammatory Fibrosis in Kidney Transplantation: The Banff I-IFTA Lesion. Am J Transplant (2018) 18(2):364–76. doi: 10.1111/ajt.14609 29194971

[4] Matas AJ Helgeson ES Gaston R Cosio F Mannon R Kasiske BL. Inflammation in Areas of Fibrosis: The DeKAF Prospective Cohort. Am J Transplant (2020) 20(9):2509–21. doi: 10.1111/ajt.15862 32185865

[5] Nankivell BJ Agrawal N Sharma A Taverniti A P'Ng CH Shingde M. The Clinical and Pathological Significance of Borderline T Cell-Mediated Rejection. Am J Transplant (2019) 19(5):1452–63. doi: 10.1111/ajt.15197 30501008

[6] Loupy A Haas M Roufosse C Naesens M Adam B Afrouzian M. The Banff 2019 Kidney Meeting Report (I): Updates on and Clarification of Criteria for T Cell- and Antibody-Mediated Rejection. Am J Transplant (2020) 20(9):2318–31. doi: 10.1111/ajt.15898 PMC7496245 32463180

[7] Swanson KJ Aziz F Garg N Mohamed M Mandelbrot D Djamali A. Role of Novel Biomarkers in Kidney Transplantation. World J Transplant (2020) 10(9):230–55. doi: 10.5500/wjt.v10.i9.230 PMC7504189 32995319

[8] Crespo E Roedder S Sigdel T Hsieh SC Luque S Cruzado JM. Molecular and Functional Noninvasive Immune Monitoring in the ESCAPE Study for Prediction of Subclinical Renal Allograft Rejection. Transplantation (2017) 101(6):1400–9. doi: 10.1097/TP.0000000000001287 27362314

[9] Montero N Farouk S Gandolfini I Crespo E Jarque M Meneghini M. Pretransplant Donor-Specific Ifnγ ELISPOT as a Predictor of Graft Rejection: A Diagnostic Test Accuracy Meta-Analysis. Transplant Direct (2019) 5(5):e451. doi: 10.1097/TXD.0000000000000886 31165086 PMC6511445

[10] Schachtner T Otto NM Stein M Reinke P. Transplantectomy is Associated With Presensitization With Donor-Reactive T Cells and Graft Failure After Kidney Retransplantation: A Cohort Study. Nephrol Dial Transplant (2018) 33(5):889–96. doi: 10.1093/ndt/gfy002 29401311

[11] Roedder S Sigdel T Salomonis N Hsieh S Dai H Bestard O. The kSORT Assay to Detect Renal Transplant Patients at High Risk for Acute Rejection: Results of the Multicenter AART Study. PloS Med (2014) 11(11):e1001759. doi: 10.1371/journal.pmed.1001759 25386950 PMC4227654

[12] Friedewald JJ Kurian SM Heilman RL Whisenant TC Poggio ED Marsh C. Development and Clinical Validity of a Novel Blood-Based Molecular Biomarker for Subclinical Acute Rejection Following Kidney Transplant. Am J Transplant (2019) 19(1):98–109. doi: 10.1111/ajt.15011 29985559 PMC6387870

[13] Zhang W Yi Z Keung KL Shang H Wei C Cravedi P. A Peripheral Blood Gene Expression Signature to Diagnose Subclinical Acute Rejection. J Am Soc Nephrol (2019) 30(8):1481–94. doi: 10.1681/ASN.2018111098 PMC6683710 31278196

[14] Van de Vrie M Deegens JK Eikmans M van der Vlag J Hilbrands LB. Urinary MicroRNA as Biomarker in Renal Transplantation. Am J Transplant (2017) 17(5):1160–6. doi: 10.1111/ajt.14082 PMC5434819 27743494

[15] Lorenzen JM Schauerte C Kölling M Hübner A Knapp M Haller H. Long Noncoding RNAs in Urine Are Detectable and May Enable Early Detection of Acute T Cell-Mediated Rejection of Renal Allografts. Clin Chem (2015) 61:1505–14. doi: 10.1373/clinchem.2015.243600 26506996

[16] Oellerich M Shipkova M Asendorf T Walson PD Schauerte V Mettenmeyer N. Absolute Quantification of Donor-Derived Cell-Free DNA as a Marker of Rejection and Graft Injury in Kidney Transplantation: Results From a Prospective Observational Study. Am J Transplant (2019) 19(11):3087–99. doi: 10.1111/ajt.15416 PMC6899936 31062511

[17] Whitlam JB Ling L Skene A Kanellis J Ierino FL Slater HR. Diagnostic Application of Kidney Allograft-Derived Absolute Cell-Free DNA Levels During Transplant Dysfunction. Am J Transplant (2019) 19(4):1037–49. doi: 10.1111/ajt.15142 30312536

[18] Halloran PF Reeve J Akalin E Aubert O Bohmig GA Brennan D. Real Time Central Assessment of Kidney Transplant Indication Biopsies by Microarrays: The INTERCOMEX Study. Am J Transplant (2017) 17(11):2851–62. doi: 10.1111/ajt.14329 28449409

[19] Seifert ME Yanik MV Feig DI Hauptfeld-Dolejsek V Mroczek-Musulman EC Kelly DR. Subclinical Inflammation Phenotypes and Long-Term Outcomes After Pediatric Kidney Transplantation. Am J Transplant (2018) 18(9):2189–99. doi: 10.1111/ajt.14933 PMC6436389 29766640

[20] Kee TY Chapman JR O'Connell PJ Fung CL Allen RD Kable K. Treatment of Subclinical Rejection Diagnosed by Protocol Biopsy of Kidney Transplants. Transplantation (2006) 82(1):36–42. doi: 10.1097/01.tp.0000225783.86950.c2 16861939

[21] Nankivell BJ Borrows RJ Fung CL O'Connell PJ Allen RD Chapman JR. Natural History, Risk Factors, and Impact of Subclinical Rejection in Kidney Transplantation. Transplantation (2004) 78(2):242–9. doi: 10.1097/01.TP.0000128167.60172.CC 15280685

[22] Stites E Kumar D Olaitan O John Swanson S Leca N Weir M. High Levels of dd-cfDNA Identify Patients With TCMR 1A and Borderline Allograft Rejection at Elevated Risk of Graft Injury. Am J Transplant (2020) 20(9):2491–8. doi: 10.1111/ajt.15822 PMC7496411 32056331

[23] Geneugelijk K Niemann M Drylewicz J Zuilen AD Joosten I Allebes WA. PIRCHE-II Is Related to Graft Failure After Kidney Transplantation. Front Immunol (2018) 9:321. doi: 10.3389/fimmu.2018.00321 29556227 PMC5844930

[24] Lachmann N Niemann M Reinke P Budde K Schmidt D Halleck F. Donor-Recipient Matching Based on Predicted Indirectly Recognizable HLA Epitopes Independently Predicts the Incidence of *De Novo* Donor-Specific HLA Antibodies Following Renal Transplantation. Am J Transplant (2017) 17(12):3076–86. doi: 10.1111/ajt.14393 28613392

[25] Naef B Nilsson J Wuethrich RP Mueller TF Schachtner T. Intravenous Immunoglobulins Do Not Prove Beneficial to Reduce Alloimmunity Among Kidney Transplant Recipients With BKV-Associated Nephropathy. Transpl Int (2021) 34(8):1481–93. doi: 10.1111/tri.13882 33872427

[26] Mehta R Bhusal S Randhawa P Sood P Cherukuri A Wu C. Short-Term Adverse Effects of Early Subclinical Allograft Inflammation in Kidney Transplant Recipients With a Rapid Steroid Withdrawal Protocol. Am J Transplant (2018) 18(7):1710–7. doi: 10.1111/ajt.14627 29247472

[27] Bouatou Y Viglietti D Pievani D Louis K Duong Van Huyen JP Rabant M. Response to Treatment and Long-Term Outcomes in Kidney Transplant Recipients With Acute T Cell-Mediated Rejection. Am J Transplant (2019) 19(7):1972–88. doi: 10.1111/ajt.15299 30748089

[28] Sood P Cherikh WS Toll AE Mehta RB Hariharan S. Kidney Allograft Rejection: Diagnosis and Treatment Practices in USA- A UNOS Survey. Clin Transplant (2021) 35(4):e14225. doi: 10.1111/ctr.14225 33455009

[29] Bloom RD Bromberg JS Poggio ED Bunnapradist S Langone AJ Sood P. Circulating Donor-Derived Cell-Free DNA in Blood for Diagnosing Active Rejection in Kidney Transplant Recipients (DART) Study Investigators. Cell-Free DNA and Active Rejection in Kidney Allografts. J Am Soc Nephrol (2017) 28(7):2221–32. doi: 10.1681/ASN.2016091034 PMC5491290 28280140

[30] Huang E Sethi S Peng A Najjar R Mirocha J Haas M. Early Clinical Experience Using Donor-Derived Cell-Free DNA to Detect Rejection in Kidney Transplant Recipients. Am J Transplant (2019) 19(6):1663–70. doi: 10.1111/ajt.15289 30725531

[31] Unterrainer C Döhler B Niemann M Lachmann N Süsal C. Can PIRCHE-II Matching Outmatch Traditional HLA Matching? Front Immunol (2021) 12:631246. doi: 10.3389/fimmu.2021.631246 33717167 PMC7952296

[32] Crespo E Cravedi P Martorell J Luque S Melilli E Cruzado JM. Posttransplant Peripheral Blood Donor-Specific Interferon-γ Enzyme-Linked Immune Spot Assay Differentiates Risk of Subclinical Rejection and *De Novo* Donor-Specific Alloantibodies in Kidney Transplant Recipients. Kidney Int (2017) 92(1):201–13. doi: 10.1016/j.kint.2016.12.024 PMC5930006 28274484

[33] Meneghini M Crespo E Niemann M Torija A Lloberas N Pernin V. Donor/Recipient HLA Molecular Mismatch Scores Predict Primary Humoral and Cellular Alloimmunity in Kidney Transplantation. Front Immunol (2021) 11:623276. doi: 10.3389/fimmu.2020.623276 33776988 PMC7988214

[34] Siu JHY Surendrakumar V Richards JA Pettigrew GJ. T Cell Allorecognition Pathways in Solid Organ Transplantation. Front Immunol (2018) 9:2548. doi: 10.3389/fimmu.2018.02548 30455697 PMC6230624

[35] Wiebe C Rush DN Gibson IW Pochinco D Birk PE Goldberg A. Evidence for the Alloimmune Basis and Prognostic Significance of Borderline T Cell-Mediated Rejection. Am J Transplant (2020) 20(9):2499–508. doi: 10.1111/ajt.15860 PMC7496654 32185878

[36] Tomosugi T Iwasaki K Sakamoto S Niemann M Spierings E Nahara I. Clinical Significance of Shared T Cell Epitope Analysis in Early *De Novo* Donor-Specific Anti-HLA Antibody Production After Kidney Transplantation and Comparison With Shared B Cell Epitope Analysis. Front Immunol (2021) 12:621138. doi: 10.3389/fimmu.2021.621138 33897684 PMC8061417

[37] Geneugelijk K Spierings E. PIRCHE-II: An Algorithm to Predict Indirectly Recognizable HLA Epitopes in Solid Organ Transplantation. Immunogenetics (2020) 72(1-2):119–29. doi: 10.1007/s00251-019-01140-x PMC6971131 31741009

[38] Ekberg H Tedesco-Silva H Demirbas A Vítko S Nashan B Gürkan A. ELITE-Symphony Study. Reduced Exposure to Calcineurin Inhibitors in Renal Transplantation. N Engl J Med (2007) 357(25):2562–75. doi: 10.1056/NEJMoa067411 18094377

[39] Nickel P Presber F Bold G Biti D Schönemann C Tullius SG. Enzyme-Linked Immunosorbent Spot Assay for Donor-Reactive Interferon-Gamma-Producing Cells Identifies T-Cell Presensitization and Correlates With Graft Function at 6 and 12 Months in Renal-Transplant Recipients. Transplantation (2004) 78(11):1640–6. doi: 10.1097/01.TP.0000144057.31799.6A 15591953

