# Supplementary material for: High PIRCHE Scores May Allow Risk Stratification of Borderline Rejection in Kidney Transplant Recipients
Source: Front Immunol. 2022 Feb 18;13:788818. doi: 10.3389/fimmu.2022.788818 (PMC8894244; doi:10.3389/fimmu.2022.788818)
Supplement: Supplementary file 1 [file DataSheet_1.docx]

**Supplement Table 1A.** Clinical Characteristics of KTRs with stable/impaired allograft function.

|  | **Stable function**  **(n=37)** | **Impaired function >20% (n=23)** | ***P value*** |
| --- | --- | --- | --- |
| **Recipient Characteristics** |  |  |  |
| Recipient age, years* | 56 (19-74) | 58 (28-72) | *0.837* |
| Renal disease, n (%) |  |  |  |
| Diabetic  Hypertensive  PKD  Glomerular disease  Others/unknown | 3 (8.1%)  2 (5.4%)  5 (13.5%)  15 (40.5%)  12 (32.5%) | 4 (17.4%)  2 (8.7%)  2 (8.7%)  9 (39.1%)  6 (26.1%) | *0.326* |
| Recipient, male sex, n (%) | 24 (64.86%) | 11 (47.8%) | *0.282* |
| Deceased donation, n (%) | 28 (75.7%) | 18 (78.3%) | *1* |
| Living donation, n (%) | 9 (24.3%) | 5 (21.7%) | *1* |
| AB0 incompatible, n (%) | 1 (2.7%) | 1 (4.3%) | *1* |
| Retransplantation, n (%) | 4 (10.8%) | 10 (43.5%) | *0.005*** |
| Cold ischemia time, minutes* | 609 (312-1197) | 553 (329-1195) | *0.710* |
| **Immunosuppression** |  |  |  |
| Induction IS, n (%)  Lymphocyte depletion  IL-2 receptor blockade  AB0 desensitization  Other | 15 (40.5%)  20 (54.1%)  1 (2.7%)  1 (2.7%) | 8 (34.8%)  14 (60.8%)  1 (4.4%)  0 | *0.715* |
| Maintenance IS, n (%)  Tacrolimus  Ciclosporine  MMF/MPA  Azathioprin | 28 (75.7%)  9 (24.3%)  36 (96.3%)  1 (2.7%) | 21 (91.30%)  2 (8.69%)  23 (100%)  0 | *0.178*  *0.178*  *1*  *1* |
| Steroid free at 1 year, n (%) | 15 (40.5%) | 13 (56.5%) | *0.291* |
| **Donor Characteristics** |  |  |  |
| Donor age, years | 57 (0-78) | 52 (31-69) | *0.643* |
| Donor male sex, n (%) | 20 (54.1%) | 9 (39.13%) | *0.298* |
| **Immunocompatibility** |  |  |  |
| Total HLA Mismatches* | 7 (2-10) | 7 (2-8) | *0.393* |
| Total PIRCHE-Score*  PIRCHE-A*  PIRCHE-B*  PIRCHE-C*  PIRCHE-DR*  PIRCHE-DQ* | 86.93 (17.37-195.43)  14.00 (0-69.37)  13.37 (0.23-44.12)  13.00 (0-32.73)  17.57 (0-56.13)  23.32 (0-60.13) | 76.62 (23.60-163.98)  14.89 (0.02-45.78)  18.34 (0-44.63)  14.77 (1.13-37.09)  13.02 (0.14-33.34)  22.84 (0-48.84) | *0.750*  *0.632*  *0.744*  *0.627*  *0.466*  *0.406* |
| Preformed DSA, n (%)^§^ | 7 (16.2%) | 11 (47.8%) | *0.023** |

* median (range)

^§^ Preformed DSA: DSA against the current renal graft with MFI > 500 at any time before the transplantation, but latest MFI <1000 prior to transplantation

**Supplement Table 1B.** Outcomes in KTRs with with stable/impaired allograft function.

|  | **Stable function**  **(n=37)** | **Impaired function >20% (n=23)** | ***P value*** |
| --- | --- | --- | --- |
| **Allograft function** |  |  |  |
| DGF (%) | 11 (29.7%) | 1 (4.3%) | *0.020** |
| Creatinine baseline before biopsy, µmol/L* | 152 (80-649) | 93 (54-169) | *-* |
| Creatinine at biopsy, µmol/L* | 187 (87-613) | 148 (92-287) | *-* |
| Creatinine baseline 1^st^ year after biopsy, µmol/L* | 125 (73-597) | 135 (97-230) | *-* |
| **Allograft biopsy** |  |  |  |
| Time after transplantation, months* | 2 (0-64) | 14 (2-74) | *<0.001**** |
| < 6 months, n (%)  6-12 months, n (%)  13 - 60 months, n (%)  > 60 months, n (%) | 30 (81.1%)  4 (10.8%)  2 (5.4%)  1 (2.7%) | 4 (17.5%)  7 (30.4%)  11 (47.8%)  1 (4.3%) | *<0.001**** |
| Indication for biopsy |  |  |  |
| Protocol, n (%)  eGFR/Proteinuria, n (%)  BKV replication, n (%)  DSA, n (%) | 2 (5.4%)  33 (89.2%)  1 (2.7%)  1 (2.7%) | 1 (4.3%)  19 (82.7%)  2 (8.7%)  0 | *0.550* |
| **Biopsy Findings** |  |  |  |
| Borderline only, n (%)  Borderline and ATN, n (%)  Borderline and arteriolar hyalinosis, n (%)  Borderline and other^†^, n (%) | 9 (24.3%)  10 (27.1%)  1 (2.7%)  17 (45.9%) | 7 (30.4%)  5 (21.73%)  4 (17.5%)  7 (30.4%) | *0.181* |
| i0 t≥1, n (%) | 31 (83.8%) | 20 (86.95%) | *1* |
| i0 t1, n (%)  i0 t2, n (%)  i0 t3, n (%) | 27 (73%)  3 (8.1%)  1 (2.7%) | 16 (69.6%)  2 (8.7%)  2 (8.7%) | *0.600* |
| i≥1 t≥1, n (%) | 6 (16.2%) | 3 (13.1%) | *1* |
| i1 t1, n (%)  i1 t2, n (%)  i1 t3, n (%)  i2 t1, n (%)  i3 t1, n (%) | 3 (8.1%)  2 (5.4%)  1 (2.7%)  n/a  n/a | 1 (4.3%)  1 (4.3%)  0  0  1 (4.3%) | *0.707* |
| **De novo DSA, n (%)**^#^ | 8 (21.6%) | 4 (17.5%) | *0.752* |
| IgG Class I, n (%) | 3 (8.1%) | 1 (4.3%) | *-* |
| IgG Class II, n (%) | 6 (16.2%) | 3 (13.1%) | *-* |
| **Viral infections** |  |  |  |
| BKV replication at any time, n (%)^§^ | 11 (29.7%) | 5 (21.7%) | *0.561* |
| BKV replication at time of biopsy, n (%) | 8 (21.6%) | 4 (17.4%) | *0.752* |
| CMV replication at any time, n (%) | 19 (51.35%) | 15 (65.2%) | *0.422* |
| CMV replication at time of biopsy, n (%) | 6 (16.2%) | 5 (21.73%) | *0.734* |
| **Rejection in follow-up biopsy, n (%)** | 5 (13.5%) | 3 (13.1%) | *1* |
| TCMR 1A and 1B, n (%) | 1 (2.7%) | 0 | *1* |
| TCMR 2A and 2B, n (%) | 4 (10.8%) | 2 (4.3%) | *1* |
| ABMR, n (%) | 0 | 1 (4.3%) | *0.383* |

* median (range)

^†^ Other: recurrence of renal disease (1 KTR with FSGS, 3 KTRs with IgAN), 3 KTRs with CNI-associated toxicity, 1 KTR with interstitial fibrosis, 2 KTRs with TMA, 2 KTS with necrotic lesions, 2 KTRs with obstruction, 3 KTRs with pyelonephritis, 1 KRT with adenovirus-associated acute tubulointerstitial nephritis, 1 KTR with oxalate crystals, 2 KTRs with IgA-positivity with unclear clinical significance, 1 KTR with presumable Foscarnet-associated changes

^#^ 1 KTR has developed de novo DSA for both class I and II

**Supplement Table 2A.** Clinical characteristics of KTRs with/without de novo DSA.

|  | **No de novo DSA**  **(n=48)** | **De novo DSA**  **(n=12)** | ***P value*** |
| --- | --- | --- | --- |
| **Recipient Characteristics** |  |  |  |
| Recipient age, years* | 56.5 (19-72) | 57 (28-74) | *0.618* |
| Renal disease, n (%) |  |  |  |
| Diabetic  Hypertensive  PKD  Glomerular disease  Others/unknown | 6 (12.5%)  4 (8.3%)  5 (10.4%)  17 (35.4%)  16 (33.3%) | 1 (8.3%)  0  2 (16.7%)  7 (58.3%)  2 (16.7%) | *0.353* |
| Recipient, male sex, n (%) | 27 (56.2%) | 8 (66.7%) | *0.745* |
| Deceased donation, n (%) | 35 (72.9%) | 11 (91.7%) | *0.261* |
| Living donation, n (%) | 13 (27.1%) | 1 (8.3%) | *0.261* |
| AB0 incompatible, n (%) | 2 (4.2%) | 0 | *1* |
| Retransplantation, n (%) | 11 (22.9%) | 3 (25%) | *1* |
| Cold ischemia time, minutes* | 570 (312-1197) | 528 (329-1195) | *0.857* |
| **Immunosuppression** |  |  |  |
| Induction IS, n (%)  Lymphocyte depletion  IL-2 receptor blockade  AB0 desensitization  Other | 15 (31.2%)  30 (62.5%)  2 (4.2%)  1 (2.1%) | 8 (66.7%)  4 (33.3%)  0  0 | *0.115* |
| Maintenance IS, n (%)  Tacrolimus  Ciclosporine  MMF/MPA  Azathioprin | 42 (87.5%)  6 (12.5%)  47 (97.9%)  1 (2.1%) | 7 (58.8%)  5 (41.2%)  12 (100%)  0 | *0.033**  *0.033**  *1*  *1* |
| Steroid free at 1 year, n (%) | 21 (43.7%) | 7 (58.3%) | *0.520* |
| **Donor Characteristics** |  |  |  |
| Donor age, years* | 55.5 (0-78) | 57 (34-74) | *0.912* |
| Donor male sex, n (%) | 24 (50.0%) | 5 (41.7%) | *0.750* |
| **Immunocompatibility** |  |  |  |
| Total HLA Mismatches* | 7 (2-10) | 8 (4-10) | *0.282* |
| Total PIRCHE-Score*  PIRCHE-A*  PIRCHE-B*  PIRCHE-C*  PIRCHE-DR*  PIRCHE-DQ* | 83.85 (17.37-195.43)  13.18 (0-69.37)  16.6 (0-44.63)  14.75 (0-37.09)  15.78 (0-56.13)  23.64 (0-60.13) | 89.45 (43.79-142.61)  18.0 (0-41.0)  12.2 (3-30.03)  11.84 (0-28.2)  12.44 (3-24.0)  22.78 (8.62-47.34) | *0.760*  *0.651*  *0.360*  *0.346*  *0.637*  *0.992* |
| Preformed DSA, n (%)^§^ | 14 (29.2%) | 4 (33.3%) | *0.740* |

* median (range)

^§^ Preformed DSA: DSA against the current renal graft with MFI > 500 at any time before the transplantation, but latest MFI <1000 prior to transplantation

**Supplement Table 2B.** Outcomes of KTRs with/without de novo DSA.

|  | **No de novo DSA**  **(n= 48)** | **De novo DSA**  **(n=12)** | ***P value*** |
| --- | --- | --- | --- |
| DGF, n (%) | 7 (14.6%) | 5 (41.7%) | *0.051* |
| **Allograft biopsy** |  |  |  |
| Time after transplantation, months* | 3.5 (0-74) | 6 (0-64) | *0.451* |
| < 6 months, n (%)  6-12 months, n (%)  13 - 60 months, n (%)  > 60 months, n (%) | 28 (58.3%)  9 (18.8%)  10 (20.8%)  1 (2.1%) | 6 (50%)  2 (16.7%)  3 (25%)  1 (8.3%) | *0.719* |
| Indication for biopsy |  |  |  |
| Protocol, n (%)  eGFR/ Proteinuria, n (%)  BKV replication, n (%)  DSA°, n (%) | 3 (6.2%)  41 (85.4%)  2 (4.2%)  2 (4.2%) | 0  11 (91.7%)  0  1 (8.3%) | *0.453* |
| **Biopsy Findings** |  |  |  |
| Borderline only, n (%)  Borderline and ATN, n (%)  Borderline and arteriolar hyalinosis, n (%)  Borderline and other^†^, n (%) | 13 (27.2%)  10 (20.8%)  3 (6.2%)  22 (45.8%) | 3 (25%)  5 (41.7%)  2 (16.7%)  2 (16.7%) | *0.173* |
| i0 t≥1, n (%) | 40 (83.3%) | 11 (91.7%) | *0.671* |
| i0 t1, n (%)  i0 t2, n (%)  i0 t3, n (%) | 33 (68.7%)  4 (8.3%)  3 (6.2%) | 10 (83.4%)  1 (8.3%)  0 | *0.460* |
| i≥1 t≥1, n (%) | 8 (16.7%) | 1 (8.3%) | *0.671* |
| i1 t1, n (%)  i1 t2, n (%)  i1 t3, n (%)  i2 t1, n (%)  i3 t1, n (%) | 4 (8.3%)  3 (6.2%)  0  0  1 (2.1%) | 0  0  1 (8.3%)  0  0 | *0.013** |
| **De novo DSA, n (%)**^#^ | n/a | 12 (100%) | *-* |
| IgG Class I, n (%) | n/a | 4 (33.3%) | *-* |
| IgG Class II, n (%) | n/a | 9 (75%) | *-* |
| **Viral infections** |  |  |  |
| BKV replication at any time, n (%) | 12 (25%) | 4 (33.3%) | *0.716* |
| BKV replication at time of biopsy, n (%) | 10 (20.8%) | 2 (16.7%) | *1* |
| CMV replication at any time, total n (%) | 27 (56.2%) | 7 (58.3%) | *1* |
| CMV replication at time of biopsy, n (%) | 11 (22.9%) | 0 | *0.099* |
| **Rejection in follow-up biopsy, n (%)** | 5 (10.4%) | 3 (25%) | *0.191* |
| TCMR 1A and 1B, n (%) | 0 | 1 (8.3%) | *0.200* |
| TCMR 2A and 2B, n (%) | 4 (8.3%) | 2 (16.7%) | *0.590* |
| ABMR chronic active, n (%) | 1 (2.1%) | 0 | *1* |

* median (range)

^†^ Other: recurrence of renal disease (1 KTR with FSGS, 3 KTRs with IgAN), 3 KTRs with CNI-associated toxicity, 1 KTR with interstitial fibrosis, 2 KTRs with TMA, 2 KTS with necrotic lesions, 2 KTRs with obstruction, 3 KTRs with pyelonephritis, 1 KRT with adenovirus-associated acute tubulointerstitial nephritis, 1 KTR with oxalate crystals, 2 KTRs with IgA-positivity with unclear clinical significance, 1 KTR with presumable Foscarnet-associated changes

^#^ 1 KTR has developed de novo DSA for both class I and II

**Supplement Table 3A.** Clinical Characteristics of KTRs with “i0 t1” as minimal lesion according to Banff 2018 compared to KTRs with “i1 t1” as minimal lesion according to Banff 2019.

|  | **i = 0**  **(n=51)** | **i ≥ 1**  **(n=9)** | ***P value*** |
| --- | --- | --- | --- |
| **Recipient Characteristics** |  |  |  |
| Recipient age, years* | 57 (19-72) | 54 (35-74) | *0.917* |
| Renal disease, n (%) |  |  |  |
| Diabetic  Hypertensive  PKD  Glomerular disease  Others/unknown | 5 (9.8%)  4 (7.8%)  6 (11.76%)  22 (43.1%)  14 (27.5%) | 2 (22.2%)  0  1 (11.1%)  2 (22.2%)  4 (44.4%) | *0.421* |
| Recipient, male sex, n (%) | 30 (58.82%) | 5 (55.5%) | *1* |
| Deceased donation, n (%) | 39 (76.47%) | 7 (77.8%) | *1* |
| Living donation, n (%) | 12 (23.5%) | 2 (22.2%) | *1* |
| AB0 incompatible, n (%) | 1 (2%) | 1 (11.1%) | *0.280* |
| Retransplantation, n (%) | 12 (23.5%) | 2 (22.2%) | *1* |
| Cold ischemia time, minutes* | 560 (312-1197) | 624 (336-1112) | *0.359* |
| **Immunosuppression** |  |  |  |
| Induction IS, n (%)  Lymphocyte depletion  IL-2 receptor blockade  AB0 desensitization  Other | 19 (37.25%)  31 (60.78%)  1 (1.96%)  0 | 4 (44.4%)  3 (33.3%)  1 (11.1%)  1 (11.1%) | *0.053* |
| Maintenance IS, n (%)  Tacrolimus  Ciclosporine  MMF/MPA  Azathioprin | 41 (80.4%)  10 (19.6%)  50 (98.0%)  1 (2%) | 8 (88.9%)  1 (11.1%)  9 (100%)  0 | *1*  *1*  *1*  *1* |
| Steroid free at 1 year, n (%) | 24 (47.06%) | 4 (44.4%) | *1* |
| **Donor Characteristics** |  |  |  |
| Donor age, years* | 58 (0-78) | 45 (2-60) | *0.010*** |
| Donor male sex, n (%) | 24 (47.0%) | 5 (55.5%) | *0.727* |
| **Immunocompatibility** |  |  |  |
| Total HLA Mismatches* | 7 (2-10) | 6 (2-9) | *0.721* |
| Total PIRCHE-Score*  PIRCHE-A*  PIRCHE-B*  PIRCHE-C*  PIRCHE-DR*  PIRCHE-DQ* | 89.54 (20.22-195.43)  18.94 (0-69.37)  18.34 (0-44.63)  14.73 (0-37.09)  15.68 (0-56.13)  23.24 (0-60.13) | 62.52 (17.37-107.35)  5.14 (0-19.17)  9.25 (0.23-20.29)  11.10 (6.33-32.73)  10.49 (0.08-40.17)  23.00 (0-55.72) | *0.126*  *0.005***  *0.014**  *0.576*  *0.341*  *0.768* |
| Preformed DSA, n (%)^§^ | 15 (29.4%) | 3 (33.3%) | *1* |

* median (range)

^§^ Preformed DSA: DSA against the current renal graft with MFI > 500 at any time before the transplantation, but latest MFI <1000 prior to transplantation

**Supplement Table 3B**. Outcomes of KTRs with minimal lesion of “i0 t1” according to Banff 2018 compared to KTRs with minimal lesion “i1 t1” according to Banff 2019.

|  | **i = 0**  **(n=51)** | **i ≥ 1**  **(n=9)** | ***P value*** |
| --- | --- | --- | --- |
| DGF (%) | 11 (21.56%) | 1 (11.1%) | *0.671* |
| **Allograft Biopsy** |  |  |  |
| Time posttransplant, months* | 5 (0-74) | 3 (0-26) | *0.677* |
| < 6 months, n (%)  6-12 months, n (%)  13 - 60 months, n (%)  > 60 months, n (%) | 28 (54.9%)  10 (19.6%)  11 (21.6%)  2 (3.9%) | 6 (66.7%)  1 (11.1%)  2 (22.2%)  0 | *0.767* |
| Indication for biopsy |  |  |  |
| Protocol, n (%)  eGFR/Proteinuria, n (%)  BKV replication, n (%)  DSA°, n (%) | 3 (5.9%)  44 (86.3%)  2 (3.9%)  2 (3.9%) | 0  8 (88.9%)  0  1 (11.1%) | *0.488* |
| **Biopsy Findings** |  |  |  |
| Borderline only, n (%)  Borderline and ATN, n (%)  Borderline and arteriolar hyalinosis, n (%)  Borderline and other^†^, n (%) | 13 (25.5%)  13 (25.5%)  5 (9.8%)  20 (39.2%) | 3 (33.3%)  2 (22.2%)  0  4 (44.5%) | *0.595* |
| i0 t≥1, n (%) | 51 (100%) | n/a | *-* |
| i0 t1, n (%)  i0 t2, n (%)  i0 t3, n (%) | 43 (84.3%)  5 (9.8%)  3 (5.9%) | n/a  n/a  n/a | *-*  *-*  *-* |
| i≥1 t≥1, n (%) | n/a | 9 (100%) | *-* |
| i1 t1, n (%)  i1 t2, n (%)  i1 t3, n (%)  i2 t1, n (%)  i3 t1, n (%) | n/a  n/a  n/a  n/a  n/a | 4 (44.5%)  3 (33.3%)  1 (11.1%)  n/a  1 (11.1%) | *-*  *-*  *-*  *-*  *-* |
| **De novo DSA, n (%)**^#^ | 11 (21.6%) | 1 (11.1%) | *0.671* |
| IgG Class I, n (%) | 4 (7.8%) | 0 | *1* |
| IgG Class II, n (%) | 8 (15.7%) | 1 (11.1%) | *1* |
| **Viral infections** |  |  |  |
| BKV replication at any level at any time, n (%) | 13 (25.49%) | 3 (33.3%) | *0.689* |
| BKV replication at time of biopsy, n (%) | 11 (21.6%) | 1 (11.1%) | *0.671* |
| CMV replication at any time, n (%) | 30 (58.8%) | 4 (44.4%) | *0.482* |
| CMV replication at time of biopsy, n (%) | 10 (19.6%) | 1 (11.1%) | *1* |
| **Rejection in follow-up biopsy, n (%)** | 7 (13.7%) | 1 (11.1%) | *1* |
| TCMR 1A and 1B, n (%) | 1 (1.96%) | 0 | *1* |
| TCMR 2A and 2B, n (%) | 6 (11.8%) | 0 | *0.578* |
| ABMR, n (%) | 0 | 1 (11.1%) | *0.150* |

* median (range)

^†^ Other: recurrence of renal disease (1 KTR with FSGS, 3 KTRs with IgAN), 3 KTRs with CNI-associated toxicity, 1 KTR with interstitial fibrosis, 2 KTRs with TMA, 2 KTS with necrotic lesions, 2 KTRs with obstruction, 3 KTRs with pyelonephritis, 1 KRT with adenovirus-associated acute tubulointerstitial nephritis, 1 KTR with oxalate crystals, 2 KTRs with IgA-positivity with unclear clinical significance, 1 KTR with presumable Foscarnet-associated changes

^#^ 1 KTR has developed de novo DSA for both class I and II
